# Application of the Wavelet Transform on the Unusual Lightning Flashes of the Himalayan Region, Nepal

**DOI:** 10.1155/2022/2819712

**Published:** 2022-02-22

**Authors:** Pitri Bhakta Adhikari, Aashutosh Adhikari, Binod Adhikari

**Affiliations:** ^1^Department of Physics, Tri - Chandra M. Campus, Tribhuvan University, Kathmandu, Nepal; ^2^Department of Electronics and Computer Engineering, Pulchowk Campus, Tribhuvan University, Kathmandu, Nepal; ^3^Department of Physics, St. Xavier's College, Kathmandu, Nepal; ^4^Department of Physics, Patan M. Campus, Tribhuvan University, Lalitpur, Nepal

## Abstract

The unusual lightning events are characterized by a pronounced opposite-polarity pulse prior to the main electric field waveform. These electric fields are pertinent to the unusual lightning events recorded in the Himalayan region, at the height of about 1300 m above the sea level, during the premonsoon period of 2015. For the case of unusual events, a downward positive leader approaches the ground, and there is an increase in the downward-directed electric field due to which negative leaders move upwards and will try to intercept. Continuous wavelet transform has been used to understand the features of different events of lightning frequency. The wavelet transform of different events of unusual signals in the present study reveals that the unusual flashes radiate in the spectral range of 1–412.5 kHz in the initial stage and 0.9375–337.5 kHz in the overshoot which conclude that they radiate in very low frequency to very high frequency indicating that they are composed of many microdischarges and low frequencies indicating that they are composed of very long discharges. It is believed that this research work will be useful to design engineers for installing appropriate protective measures on the appliances in the Himalayan regions.

## 1. Introduction

The measurements of electromagnetic fields due to lightning radiation have been extensively carried out from the second half of the twentieth century. The electromagnetic field is one of the simple and important tools among the measurement of lightning. To understand the lightning phenomena, the knowledge of frequency spectra is also very important. It has been suggested that the spectra of the fast transitions in the return strokes, leader steps, and intracloud discharges are believed to be very similar in both shape and amplitude of about 2 MHz [1]. The physics of causes of these features in different discharge process may be very similar. Although the information of time-domain electric field signatures of positive return strokes is available in the literature, the frequency-domain information is scarce. The frequency spectrum of the electromagnetic field generated by the lightning is of much interest for both engineering assessments and scientific investigations. The knowledge of frequency spectra is essential for scientific investigations in understanding the physical processes that take place during different lightning activities and for engineering assessments in designing the adequate lightning protection schemes to physical structures [2]. Previous studies suggested that the frequency range of 1 MHz to 20 MHz is of major concern [3–5]. These ranges correspond to the natural frequencies of physical structures, particularly the dimensions of about 10 to 100 m.

The use of the solid-state devices in the airborne vehicles has increased the threat due to electromagnetic radiation from lightning [3]. The airborne vehicles coupled with the radiation of the resonant frequency can upset the system or permanently damage the electronic system [5]. Lightning discharges are the principal cause of deleterious excitations to the electronic systems [3]. The measurement of the frequency spectrum in a lightning flash has been made either by monitoring the power received at individual frequencies and then obtaining a spectrum by using the Fourier transform [6]. The unusual lighting events are the new phenomena in the field of atmosphere observed in the Himalayan region, and the continuous wavelet transform (CWT) has not been implemented till now. So, it is motivated to study about the unusual nature of the lightning phenomena by using the new tool or technique and study about some specific aspects of energy distributions. In this paper, the unusual lightning events of the Himalayan region have been studied using the continuous wavelet transform. Selection criteria for the events and instrumentation have been described in Section 2. A brief description of the methodology has been discussed in Section 3.  Section 4 presents the result and discussion. Conclusions of the entire work are discussed in Section 5.

## 2. Instrumentation

**Figure 1:**
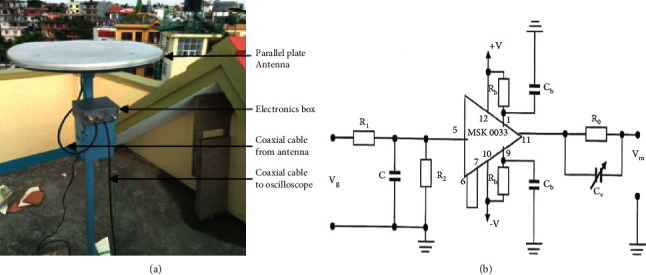
The elevated parallel plate antenna installed in Kathmandu (a) and the electronic circuit pertinent to the buffer amplifier (b).

Lightning is the electrical discharge phenomena from which the electromagnetic radiations of different wavelengths and different frequencies are being radiated. These radiations travel from the discharge channel in all possible directions, of which the vertical electric field is sensed by the parallel plate antenna. The vertical electric fields due to unusual lightning flashes were sensed by the circular flat plate antenna fixed on a 1.5 m-high post and placed on the rooftop of a building at a physical height of about 12 m from the ground as shown in Figure 1. The electric field signatures analysed in this study were measured in Himalayan region, Nepal. The upper plate of the parallel plate antenna was connected to a buffer circuit through a 60 cm-long RG-58 coaxial cable. The signal passing through the buffer amplifier was fed to PicoScope 6404D through a 20 m-long RG-58 coaxial cable. The signals so received were digitized and recorded by PicoScope at various sampling rates. It is to be noted that the parallel plate antenna that was employed for detecting and receiving the vertical electric field can detect the signal from all the directions. Unusual lightning events of different flashes were recorded in the Himalayan region of Nepal. Among the recorded flashes, only seven waveforms of different window sizes are selected, but for the sake of uniformity, a section of each record with the same window size (400 *μ*s) has been selected for the spectral features. To understand the unusual characteristics of the frequency content of different events of lightning, wavelet transform has been used in this study.

## 3. Methodology

The Fourier transform is extensively used in the field of signal processing. It is probably the most important tool for analysing signals in the electromagnetic field. The Fourier transform technique has advantages over the narrowband signal, and it has some limitations too. To understand the features of different events of lightning frequency, wavelet transform has been used. For the study of unusual lighting phenomena, the continuous wavelet transform (CWT) has been implemented. This analysis represents the energy distributions of signals in both frequency and time and makes it possible to analyze some specific aspects during unusual events [7]. Furthermore, it is useful for examining the local character of a signal that allows very precise tracking of various signals in both time and frequency, but a signal from individual components is difficult to reassemble. One major advantage afforded by CWT is the ability to perform local analysis, that is, to analyze a localized area of a larger signal. It decomposes a time-domain signal into time-frequency space. The selected transient waveforms of the electric fields of the unusual signal of lightning events have been computed. In addition, the time-domain signals and the cone of influence were also computed. A power spectrum can be calculated from the result of a wavelet transform. Then, the square modulus of the wavelet coefficient is used to provide the energy distribution in the time-scale plane. Plotting the power spectrum provides a useful graphical representation for analysing wavelet functions and for defining filters. A wavelet function can be viewed as a high-pass filter, which approximates a dataset (a signal or time series). The result of the wavelet function is the difference between the value calculated by the wavelet function and the actual data. The scaling function calculates a smoothed version of the data, which becomes the input for the next iteration of the wavelet function. Furthermore, the wavelets and scaling functions must be orthogonal to one another.

## 4. Results and Discussion

In this section, the unusual lightning events that are characterized by a pronounced opposite-polarity pulse prior to the main electric field waveform and some examples of the time-domain event and their corresponding wavelet transforms are depicted.

### 4.1. Unusual Lighting Events

**Figure 2:**
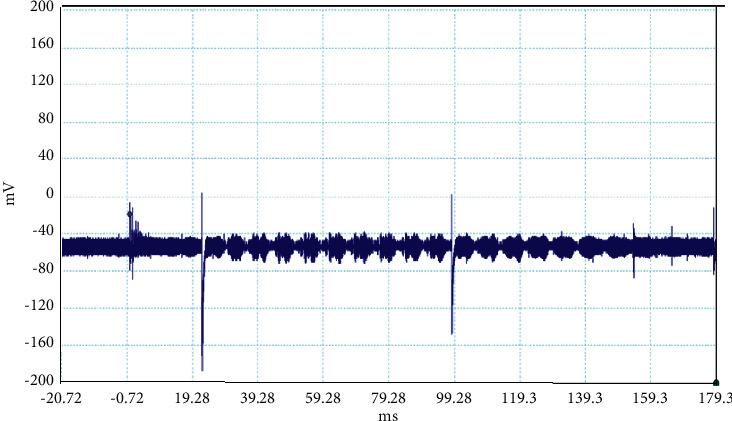
Example of the unusual lightning signal of window size 200 ms, which is recorded as a flash number 20150416-0003 in Kathmandu, Nepal.

**Figure 3:**
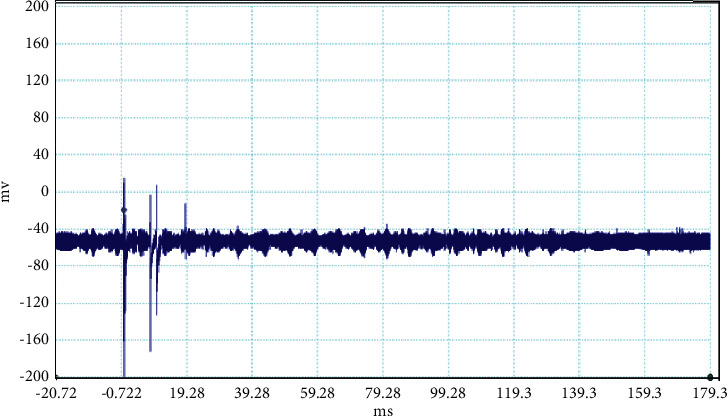
Example of the unusual lightning signal of window size 200 ms, which is recorded as a flash number 20150416-0004 in Kathmandu, Nepal.

**Figure 4:**
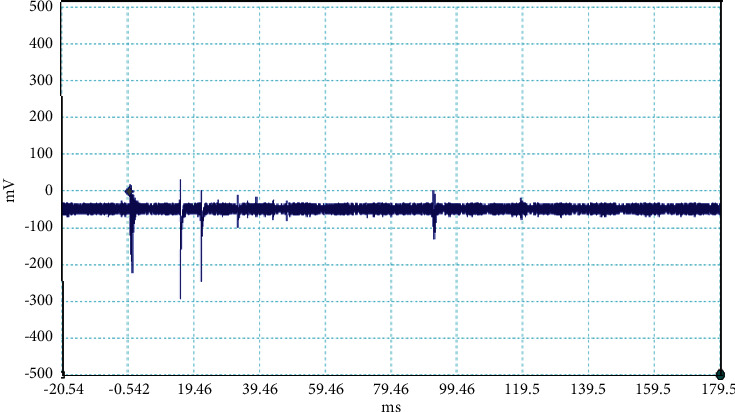
Example of the unusual lightning signal of window size 200 ms, which is recorded as a flash number 20150416-0029 in Kathmandu, Nepal.

**Figure 5:**
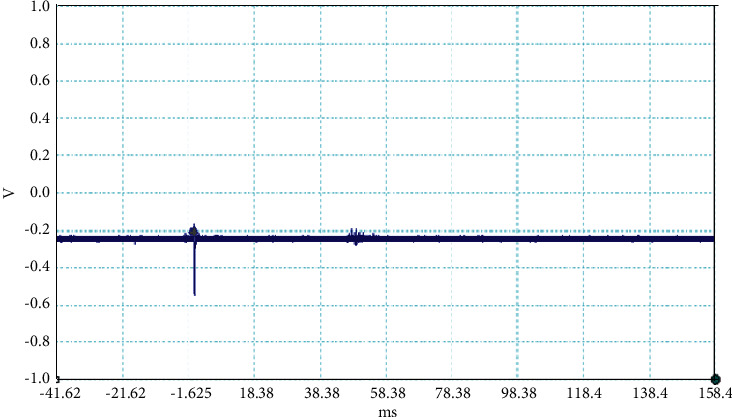
Example of the unusual lightning signal of window size 200 ms, which is recorded as a flash number 20150416-0065 in Kathmandu, Nepal.

**Figure 6:**
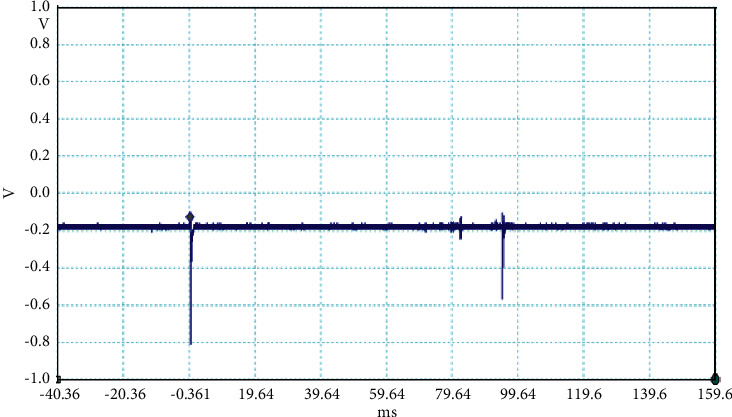
Example of the unusual lightning signal of window size 200 ms, which is recorded as a flash number 20150416-0070 in Kathmandu, Nepal.

**Figure 7:**
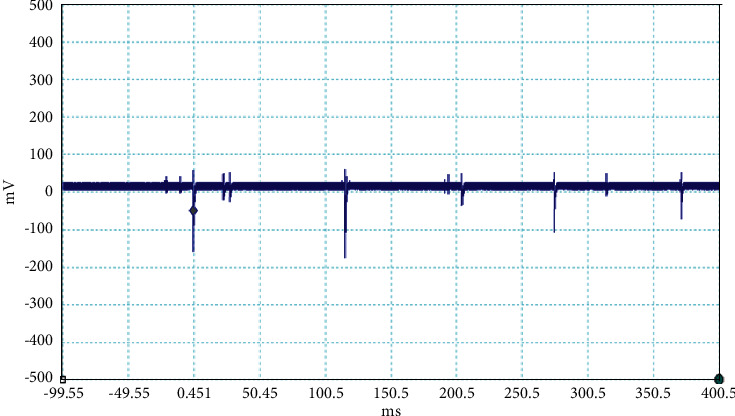
Example of the unusual lightning signal of window size 500 ms, which is recorded as a flash number 20150610-0001 in Kathmandu, Nepal.

**Figure 8:**
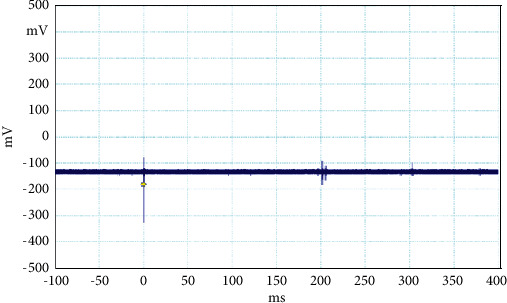
Example of the unusual lightning signal of window size 500 ms, which is recorded as a flash number 20150611-0003 in Kathmandu, Nepal.

The electrical discharge phenomenon that takes place between two charged cloud regions or between one of those regions and the ground is called lightning. The properties of lightning processes were described by fast, optical, and electromagnetic instruments. Even the new innovations were found frequently about the lightning, but still, there is mystery about it. In general, the stepped leaders move along the same direction as the main events occur, but here, in our case, the unusual lightning events are characterized by a pronounced opposite-polarity pulse prior to the main electric field waveform. This means the leader prior to the main events is in opposite nature as shown in Figures 2–8. Seven different flashes are selected from different dates, and five different flashes are selected from the date on April 16, 2015, and single waveform on each day of June 10 and June 11, 2015. These electric fields are pertinent to the unusual lightning events recorded in the Himalayan region, at the height of about 1300 m above the sea level, during the premonsoon period of 2015. For the case of unusual events, we can explain that, when a downward positive leader approaches the ground, there is an increase in the downward-directed electric field due to which negative leaders move upwards and will try to intercept. One of those upward leaders will succeed and connect, but the others may not. During this time, the unsuccessful upward negative leaders going back to the ground will produce a positive electric field change. Chen et al. studied an iterative rank-reduction method to simultaneously reconstruct the missing traces and suppress noise on spatial sampling alias and incomplete noisy data. This study demonstrates the great potential to benefit seismic investigations based on array techniques [8, 9]. In this study, the main concern has been given for the time-frequency localization using the continuous wavelet transform; however, discrete wavelet transform can be useful for a better understanding on noisy data. In addition, for a better understanding of unusual lightning phenomena, noise attenuation using local signal and noise orthogonalization can be a promising approach for the extension of this work in the near future [10].

### 4.2. Wavelet Analysis

**Figure 9:**
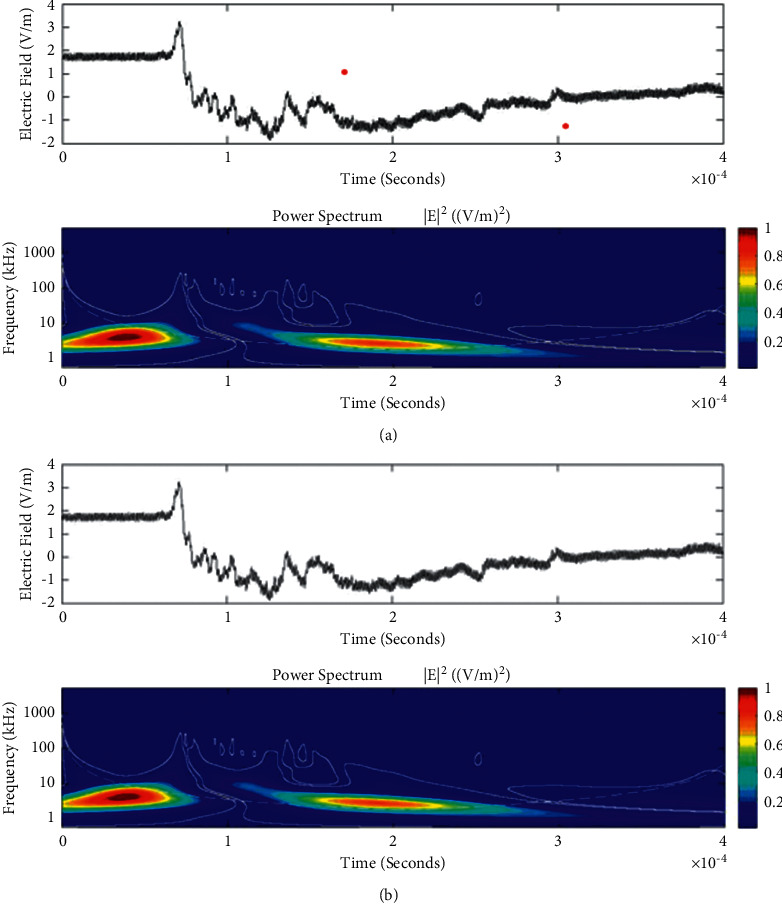
Example of the wavelet power spectrum of the first event (a) and second event (b) of the unusual lightning signal which is recorded as a flash number 20150416-0003 in Kathmandu, Nepal. The window size of the event is 400 *μ*s.

**Figure 10:**
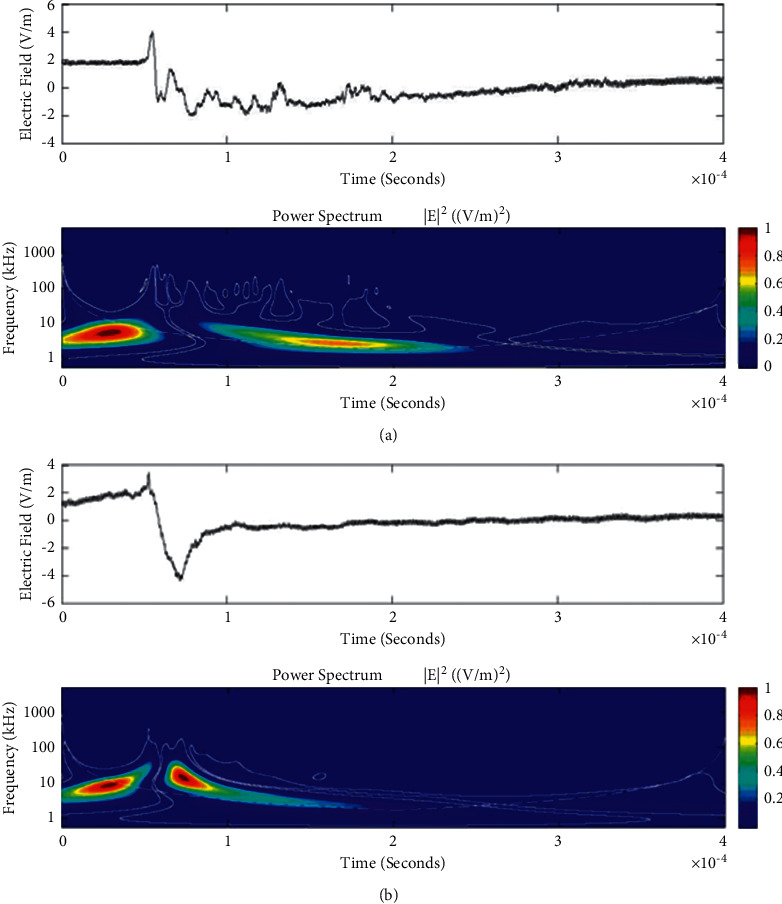
Example of the wavelet power spectrum of the first event (a) and second event (b) of the unusual lightning signal which is recorded as a flash number 20150416-0004 in Kathmandu, Nepal. The window size of the event is 400 *μ*s.

**Figure 11:**
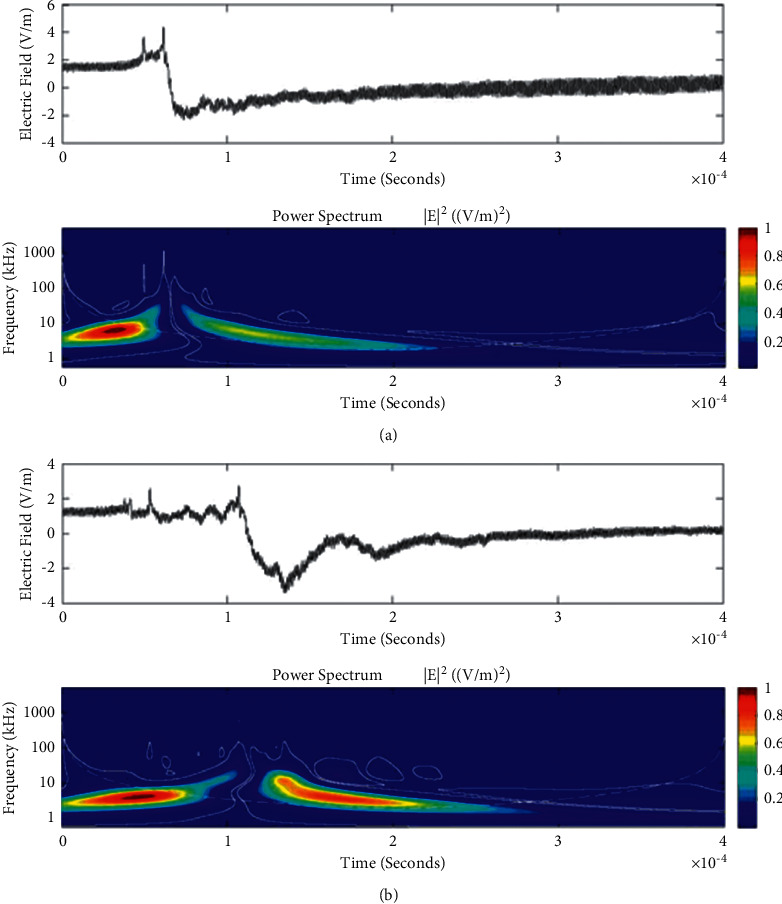
Example of the wavelet power spectrum of the first event (a) and second event (b) of the unusual lightning signal which is recorded as a flash number 20150416-0029 in Kathmandu, Nepal. The window size of the event is 400 *μ*s.

**Figure 12:**
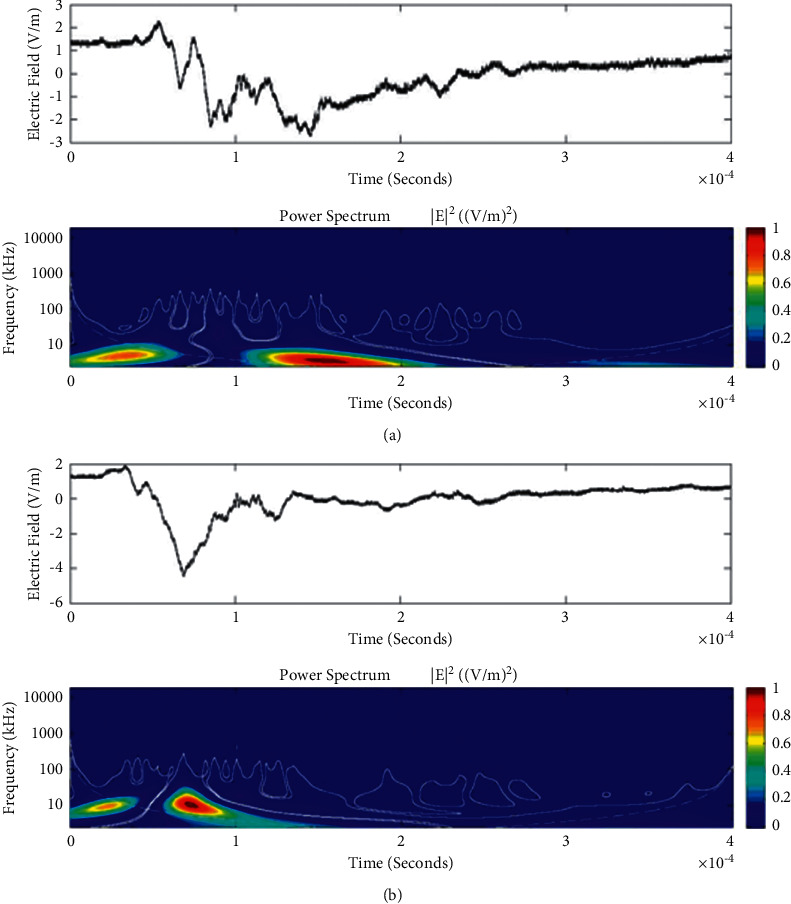
Example of the wavelet power spectrum of the first event (a) and second event (b) of the unusual lightning signal which is recorded as a flash number 20150416-0065 in Kathmandu, Nepal. The window size of the event is 400 *μ*s.

**Figure 13:**
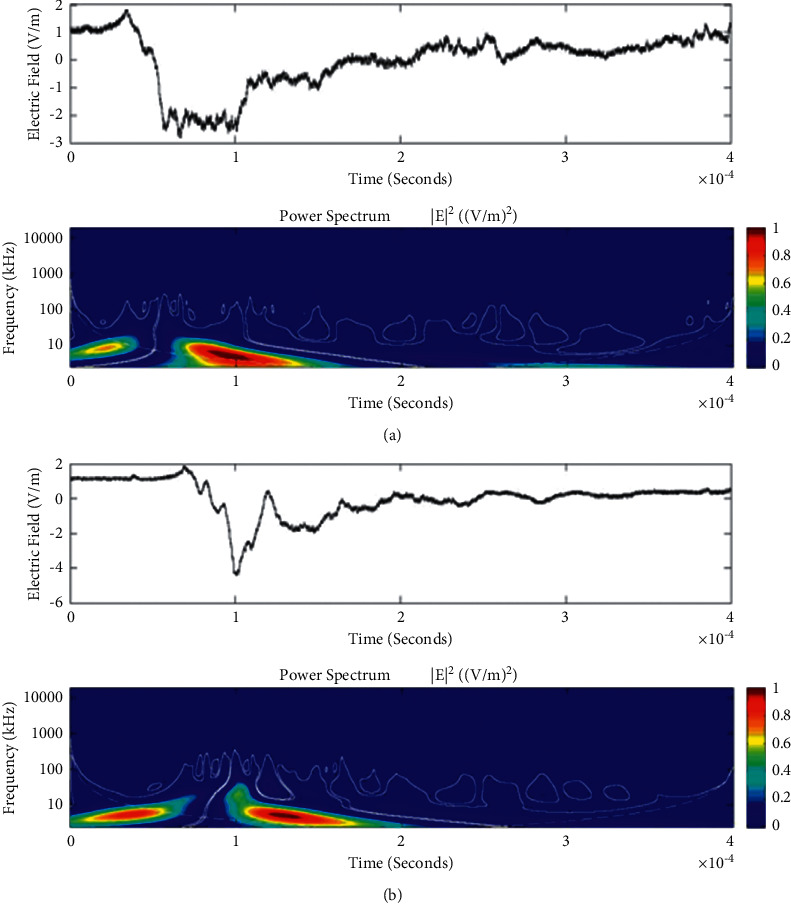
Example of the wavelet power spectrum of the first event (a) and second event (b) of the unusual lightning signal which is recorded as a flash number 20150416-0070 in Kathmandu, Nepal. The window size of the event is 400 *μ*s.

**Figure 14:**
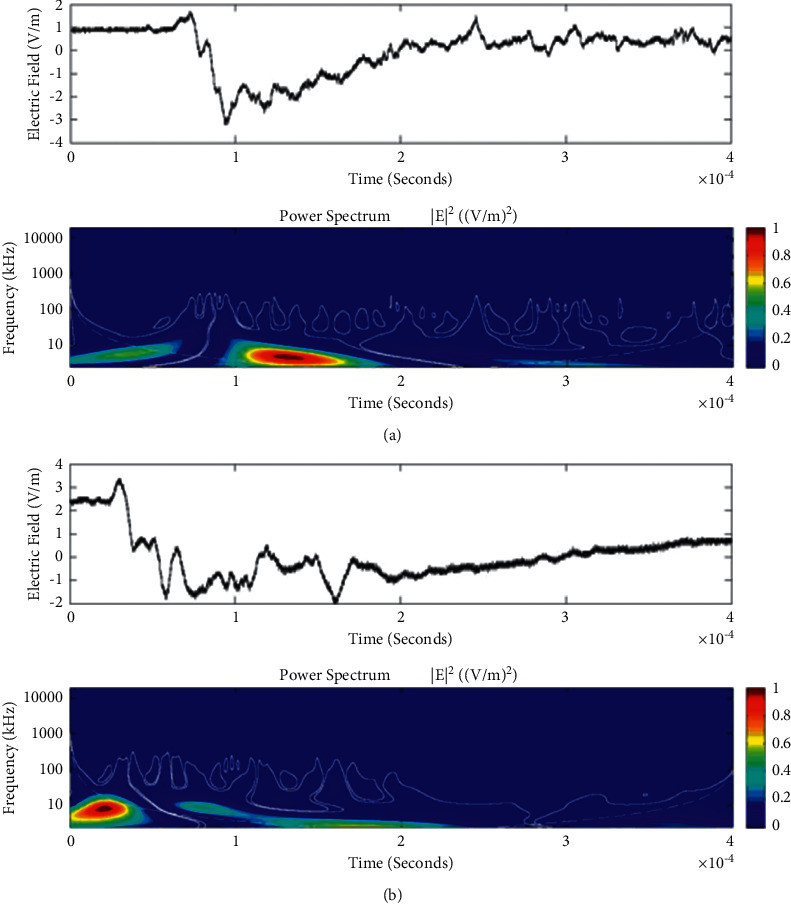
Example of the wavelet power spectrum of the first event (a) and second event (b) of the unusual lightning signal which is recorded as a flash number 20150610-0001 in Kathmandu, Nepal. The window size of the event is 400 *μ*s.

**Figure 15:**
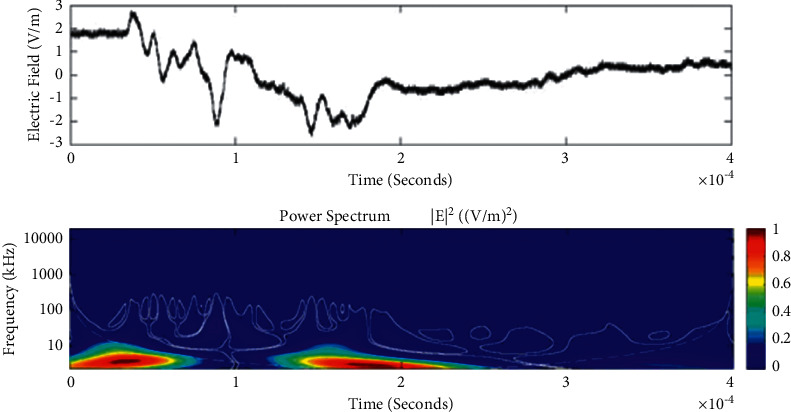
Example of the wavelet power spectrum of the event of unusual lightning signal which is recorded as a flash number 20150611-0003 in Kathmandu, Nepal. The window size of the event is 400 *μ*s.

Temporal features of different unusual lightning events have been studied using the continuous wavelet transform. Some examples of the time-domain event and their corresponding wavelet transforms are depicted in Figures 9–15. The spectral features of the unusual waveform of Figure 2 are represented in Figure 9, of Figure 3 in Figure 10, and so on, respectively. The seven different flashes, as already mentioned, are selected from different days, and the wavelet transform of these is also presented. The upper plots in each figure depict the time-domain electric fields pertinent to different events, and the lower plot depicts corresponding wavelet transforms. The vertical axis in each upper plot represents the electric field strength, whereas the left vertical axis in the lower plots represents the frequency. The horizontal axis in both plots represents the time. The magnitude of the power spectrum is represented by the colour bars along the right vertical axis of lower plots. The statistics of the wavelet power spectrum for different events are shown in Table 1 with the minimum, maximum, and average values of the spectral range, spread distribution and peak energy corresponding to the initial stage, and overshoot corresponding to each waveform. In each figure, we see spectral variations with time without the presence of continuous periodicities. The power ranges of higher intensity are seen at few different scales and different times. Since each oscillating pulse is captured as two peaks, each unusual pulse can be divided into two peaks, namely, initial peak and overshoot. The initial peak of the signals is found to radiate predominantly in the average spectral range of 1–412.5 kHz with a minimum value of 0.5 kHz to a maximum value of 1000 kHz. The spread distribution of power radiated by these pulses was found to be over an average frequency range of 5.56–81.25 kHz with a minimum value of 2 kHz to a maximum value of 500 kHz. Similarly, the overshoots of pulses are found to predominantly radiate in the average spectral range of 0.9375–337.5 kHz with a minimum value of 0.5 kHz to a maximum value of 600 kHz. The spread distribution of the power radiated by these overshoots was found to be predominant at an average frequency range of 3.44–65 kHz with a minimum value of 2 kHz to a maximum value of 100 kHz. The minimum power radiated by the initial stage is 3000, and the maximum power radiated by the initial stage is 8000. Similarly, the minimum power radiated by overshoot is 5000, and the maximum power radiated is 10000. The table depicts the power spectrum of lightning events of unusual signal waveform with the window sizes of 200 ms and 500 ms.

The wavelet spectrum also shows the occurrence of large- and small-scale features in the same time. From the figure, we see that the less intense areas are seen approximately between different scales. This result shows that periodicity is showing continuity up to lower to medium scales. However, at the period or scale above 0.5–412.5 kHz, the periodicity is noticeable, and it is seen at higher scales that areas of highest power have comparatively low periodicity. As the periodicity and the frequency have the inverse relation, the zone where the periodicity was found less is more frequent, and the zone where periodicity is high occurs less frequent. Thus, finding peak intensity with the high value at low periodicity strongly suggests temporal features of different unusual lightning events can have variations more frequently during these days. The derivative of the Gaussian wavelet algorithm was used on 200-microsecond time frame, and the power spectrum density of single and multiple peak bipolar pulses was studied by Sharma et al. [11]. Li et al. and Esa et al. used Laplace and wavelet transforms to compare different types of lightning flashes [12, 13]. The wavelet transforms have been used in various geophysical studies in the 1990s [14]; however, limited studies have been done on the analysis of lightning electromagnetic fields. The detailed study of wavelet analysis in the field of lightning activities can be found in [11, 15–23]. Thus, CWT revealed the hidden information in the unusual lighting phenomena and reflected the variation trend of the original signals on different time scales. In addition, this tool has been shown to “zoom in” to localize specific behaviours of lighting before, during, and after the unusual lightning events.

## 5. Conclusion

The unusual lightning events of the Himalayan region have been studied using the continuous wavelet transform. The unusual lightning events are characterized by a pronounced opposite-polarity pulse prior to the main electric field waveform. These electric fields are pertinent to the unusual lightning events recorded in the Himalayan region, at the height of about 1300 m above the sea level, during the premonsoon period of 2015. Continuous wavelet transform illustrated the localization of abrupt changes in both time and frequency domains. It encountered maximum fluctuations before, during, and after the unusual lightning events. During the unusual lightning time, it showed high-power short-term fluctuation; however, lower power frequency fluctuation has been observed before and after the unusual lightning time. The continuous wavelet transform worked as a powerful statistical tool which can detect fluctuations in both time and frequencies for the deeper insight into the unusual lighting events for revealing new information.

For the case of unusual events, a downward positive leader approaches the ground, and there is an increase in the downward-directed electric field due to which negative leaders move upwards and will try to intercept. Temporal features of different unusual lightning events have been studied using the continuous wavelet transform. Wavelet transform of different unusual lightning events reveals that the overshoots are the strongest source of energy at low frequency, i.e., 0.5 kHz. The peak power radiated by overshoots (8125) is higher in comparison with the initial stage (5353). The minimum frequency distribution of spectral range and spread distribution is low due to long wavelength, i.e., high window size. It is believed that this research work will be useful to design engineers for installing appropriate protective measures on the appliances in the Himalayan regions. Unlike the Fourier transform which shows the variation of power with frequency, the wavelet transform shows the most dominant frequency content of the wave. From the wavelet transform, one can easily tell the frequency localization of the maximum power radiation. Consequently, the variation of power with frequency should show maximum peaks at different frequencies for different lightning. Wavelet transform is therefore a powerful tool not only to identify the power content of the wave in time-frequency space but also to localize the power in the frequency space. Therefore, wavelet transform can be used to extract the frequency content of any signal and localize it.

## Figures and Tables

**Table 1:** Statistics of the wavelet power spectrum of the unique signal.

Statistics	Minimum	Maximum	Range
Spectral range for the initial stage (kHz)	0.5	1000	1–412.5
Spread distribution of the initial stage (kHz)	2	500	5.56–81.25
Spectral range for the overshoot (kHz)	0.5	600	0.9375–337.5
Spread distribution of the overshoot (kHz)	2	100	3.44–65
Power peak for the initial stage	3000	8000	5353
Power peak for the overshoot	5000	10000	8125

## Data Availability

The data used to support the findings of this study are available from the corresponding author upon request.
